# Preceptor support during the COVID-19 pandemic: Recommendations for continuing development

**DOI:** 10.4102/curationis.v45i1.2370

**Published:** 2022-10-27

**Authors:** Lizemari Hugo-Van Dyk, Champion N. Nyoni, Margaret Williams, Benjamin S. Botha

**Affiliations:** 1 Faculty of Health Sciences, School of Nursing, University of the Free State, Bloemfontein, South Africa; 2 Centre for Community Technology, School of Information Technology, Nelson Mandela University, Gqeberha, South Africa

**Keywords:** COVID-19, continuous professional development, clinical learning environment, preceptors, undergraduate nursing students

## Abstract

**Background:**

Mentally fit preceptors may be more capable and flexible in providing students with system, emotional and cognitive support in the clinical learning environment (CLE) in the face of any life-threatening outbreaks. Existing professional development programmes for preceptors emphasise the development of preceptor competence in a normal CLE with minimal focus on their ability to engage with adverse events that challenge their mental health.

**Objective:**

The study sought insight from preceptors’ experiences during the coronavirus disease 2019 (COVID-19) pandemic to identify their professional development programme needs while providing support to students during accompaniment.

**Method:**

A mixed methods convergent parallel design was used to collect data from 24 preceptors at a nursing education institution (NEI). Eleven preceptors responded to the survey that included the coronavirus disease 2019 (COVID-19) Stress Scale (CSS) and Burnout Assessment Tool (BAT) to collect quantitative data. Semistructured interviews were conducted with five purposively selected preceptors to collect qualitative data regarding their experiences while accompanying students during the COVID-19 pandemic.

**Results:**

Subscales within the CSS and BAT instruments were mapped against an existing preceptor support framework. Overall CSS data for each subscale indicated an average score varying from no stress to moderate stress, while BAT data shows that respondents rarely experienced burnout. However, some respondents experienced very high levels of stress and burnout. Qualitative data supplemented results.

**Conclusion:**

The COVID-19 pandemic influenced preceptors’ role in supporting students and reflecting that they amended their functioning role. Existing preceptor professional development programmes should be reviewed to ensure that the necessary concepts that foster resilience are integrated to enhance the functional role of preceptors in adversity.

**Contribution:**

Existing preceptor professional development programmes should be reviewed to ensure that the necessary concepts that foster resilience are integrated to enhance the functional role of preceptors in adversity.

## Introduction

Professional development programmes for preceptors must be responsive to preceptor needs. The coronavirus disease 2019 (COVID-19) pandemic significantly changed the nursing education landscape, and many nursing education institutions (NEIs) were unprepared for the consequences of the pandemic. Emergency remote teaching was adopted as an emergency strategy to salvage the educational programmes (Maykut et al. 2020:2261). Additionally, healthcare facilities restricted clinical placements for all healthcare students, resulting in NEIs struggling to provide nursing students with the necessary opportunities to complete their clinical learning outcomes (Leaver, Stanley & Goodwin Veenema 2022:S83). Preceptors, who are expected to support students during clinical placement, had to adjust their support strategies in alignment with the consequences of the pandemic.

**FIGURE 1:**
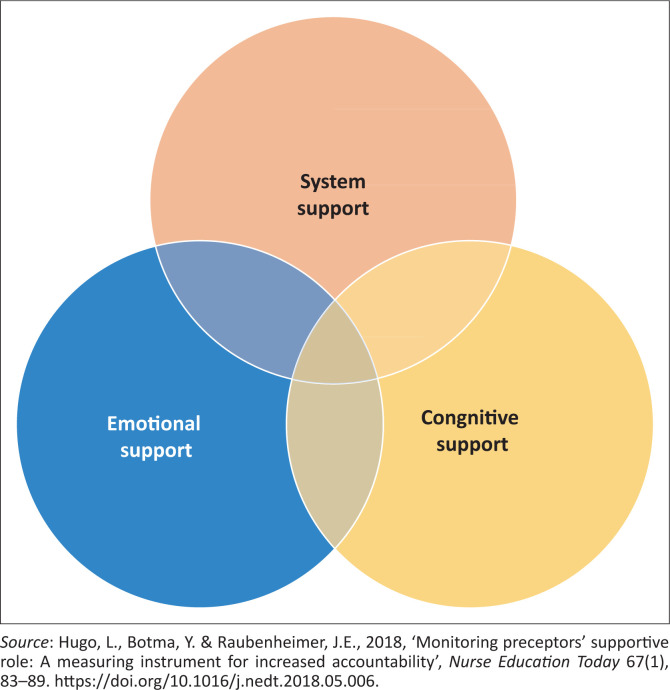
Preceptors’ support framework.

Preceptors are trained, compassionate nurse experts who develop a one-to-one time-limited relationship with a novice in the clinical setting, providing support, facilitating thinking operations and assessing competence, to promote metacognition and care that is based on the best available evidence (Botma 2014:16). Nursing education institutions employ preceptors to play a critical role in supporting nursing students, especially in undergraduate programmes. Preceptors function within the clinical platforms and may utilise skills training laboratories for the education and training of students. Hugo, Botma and Raubenheimer (2018:88) explain that students need system, emotional and cognitive support from their preceptors to integrate their theoretical knowledge into practice within the clinical learning environment (CLE) (see Figure 1). System support requires preceptors to liaise pertinent information between the NEIs and healthcare facilities, while emotional support entails them being available and showing interest in students. Cognitive support implies that preceptors facilitate the development of students’ competence while engaging in learning opportunities.

Preceptors support students in attaining their learning outcomes in the CLE. In a scoping review by Trede, Stutton and Bernoth (2016:271), preceptors were reported to assist students in gaining competence through theory–practice integration, developing professional identity and interpersonal relationships within the interprofessional team. Additionally, preceptors contribute to students’ work-readiness upon graduation, where students are exposed and supported in engaging with expectations of the world of work (Edward et al. 2017:326). The literature has demonstrated the value of preceptors among nursing students. Rambod, Sharif and Khademian (2018:444) and Hickerson, Terhaar and Taylor (2016:61) mentioned that students perceived improved learning experiences, attainment of learning outcomes and improved knowledge scores when supported by preceptors. However, most of the related research on the influence of preceptors on nursing education was not reported in adverse events such as the COVID-19 pandemic.

The COVID-19 pandemic and associated restrictions had direct implications on the role of preceptors on nursing students. The already known health worker shortage escalated, as they were infected and had to isolate. Reportedly, the increased admissions of critically ill patients and the need for continuous screening of exposed healthcare professionals exponentially increased the workload of the already compromised healthcare workers (Razu et al. 2021:2). In many settings, the healthcare system struggled to provide adequate personal protective equipment (PPE), and protocols further emphasised the risk of infection (Rose 2020:2131). Ultimately, healthcare workers, including the preceptors and the students, exhibited and reported stress, anxiety, fear of infection and helplessness due to challenges encountered in the CLE (Taylor et al. 2020:6). These issues have a direct influence on the mental health of individuals, with Despoina and Chrysoula (2020:70) reporting that mental health has a direct influence on individual functioning.

Mental health is a collective concept that integrates emotional, psychological and social well-being of individuals and has a direct impact on thinking, feelings and activities (Anicich et al. 2020:933; Despoina & Chrysoula 2020:70). A change in the environment, especially aligned with the heightened sense of doom and death, such as the COVID-19 pandemic, has been reported to negatively influence mental health (Despoina & Chrysoula 2020:75). Individuals with compromised mental health struggle to function effectively in their daily roles, further impacting expected outcomes. The COVID Stress Scale (CSS) (Taylor et al. 2020:1) and the Burnout Assessment Tool (BAT) (Schaufeli, De Witte & Desert 2020) have been applied in studies to determine the influence of the COVID-19 pandemic on the mental health of individuals. These tools determine healthcare workers’ mental state related to stress and burnout during the COVID-19 pandemic, which are necessary outcomes that influence the determination and implementation of resilience-enabling strategies (Yıldırım & Solmaz 2022:530).

## Problem statement

Mentally fit preceptors may be more capable and flexible in supporting students in the CLE in the face of any life-threatening outbreaks. Existing preceptorship professional development programmes emphasise the development of preceptor competence in the normal CLE (Hugo & Botma 2019:195) with minimal focus on their ability to engage with adverse events that challenge their mental health. Hugo and Botma (2019:195) mention that educational programmes for professional development such as the preceptorship professional development programme must be focused and driven by the needs of the preceptors.

As the role of preceptors in supporting students may have been influenced by COVID-19 and its associated restrictions, a focus on their mental health is necessary. Therefore, preceptors need to be trained on how to be mentally fit and resilient in adversity through a professional development programme that is influenced by their experiences and needs during an adverse event such as the COVID-19 pandemic. There is scant literature reporting studies exploring the influence of COVID-19 on the roles of preceptors in supporting students. This article reports on an exploratory study describing the stress and burnout experienced by preceptors during the COVID-19 pandemic while accompanying students in the CLE. Responsive professional development programmes based on the experiences of preceptors in adverse conditions could contribute to enhancing their mental health so that they can provide optimal student support.

## Research methods and design

This study used a mixed methods research design through a convergent parallel approach (Creswell 2014:135). Mixed methods research design is a powerful research approach that provides a broader spectrum of approaches to comprehensively understand a phenomenon. In this study, the outcomes of quantitative approaches through a survey using two standardised tools and a qualitative approach through semistructured interviews converged to provide an understanding of the experiences of preceptors in accompanying students in the CLE during the COVID-19 pandemic.

### Research setting

The study was conducted at an NEI offering a degree nursing programme in South Africa. The over 300 undergraduate students are placed in various CLEs, including primary healthcare, hospitals and the community. Preceptors with a degree in nursing are appointed by the NEI to accompany students during work-integrated learning. All appointed preceptors need to have completed an online professional development programme for over 12 weeks.

### Population and sampling

All 24 preceptors involved in undergraduate student accompaniment at the NEI during the time of the pandemic were included. Census sampling allowed all preceptors to be included in this study.

### Data collection

Quantitative and qualitative data were collected in two phases.

### Quantitative phase

Two standardised instruments, namely the CSS (Schaufeli et al. 2020) and the BAT (Taylor et al. 2020:1), were used for the quantitative phase.

#### COVID-19 Stress Scale

The 36-item CSS was used to measure the distress the preceptors experienced associated with COVID-19. The self-reporting instrument assesses five subscales that include danger and contamination fears, socio-economic consequences, disease-related xenophobia, compulsive checking and traumatic stress symptoms. A 5-point Likert scale required respondents to choose 1 (never), 2 (rarely), 3 (sometimes), 4 (often) and 5 (almost always) in evaluating each symptom per subscale. According to Taylor et al. (2020:4), the instrument was a good fit for the five-factor model and had an internal consistency above .80 for each subscale.

#### Burnout Assessment Tool instrument

The 33-item self-reported instrument was used to measure the burnout preceptors experienced in their workplace environment. Six core symptom dimensions were captured in the instrument and include exhaustion, emotional impairment, cognitive impairment, mental distance, psychosomatic and psychological complaints. Similar to the CCS, a 5-point Likert scale required respondents to choose 1 (never), 2 (rarely), 3 (sometimes), 4 (often) and 5 (always) in evaluating each symptom per subscale. According to Schaufeli et al. (2020), the internal consistency of the BAT was proven to be excellent with a good test-retest reliability, while the Tucker and Lewis index (0.96) and comparative fit index (0.97) proved the validity of the instrument.

Both instruments were administered electronically using Evasys (Evasys GmbH, Lüneburg, Germany). The link to the instruments and informed consent form were distributed via e-mail to all the 24 preceptors. Reminders were sent biweekly for a month. Data were collected from January 2022 to March 2022.

### Qualitative phase

During the qualitative phase, the preceptors who completed the questionnaires were invited to participate in a semistructured interview by accessing a separate Evasys link. Should they volunteer, they could click on the link to enter their details for the interviewer to arrange dates and time slots. The qualitative phase was incorporated for preceptors to elaborate on their experience of student accompaniment during the COVID-19 pandemic. A total of five preceptors indicated their interest in being interviewed.

An independent interviewer with experience in qualitative research used semistructured interviews to explore the preceptors’ experiences of the influence of the COVID-19 pandemic on their role in accompanying students. Data were collected between March 2022 and April 2022. The interview sessions lasted approximately 30–35 min. Prior to the interviews, rapport was built with preceptors to promote openness in sharing in the discussion. The interviews were conducted through open-ended questions and were recorded. The interviewer took notes and used probing to encourage a detailed exploration of experiences.

### Data analysis

Data from the CSS and BAT scales used in the quantitative phase were captured and coded using Microsoft Excel (Microsoft Corporation, Redmond, Washington, United States). Data were verified by an independent person. The average scores and standard deviations were used to interpret and describe the data of both instruments.

### COVID-19 Stress Scale analysis

The subjective rating of each item was aggregated per subscale, and subsequently, a mean was calculated across all respondents. The mean scores per subscale and that of the overall CSS were interpreted based on the CSS rating scale. For the danger and contamination subscale, a maximum of 48 points was possible, which indicated extremely high stress, 36 indicated the preceptors were very stressed and 24 meant they were moderately stressed, while a value of 12 was linked to being slightly stressed. For the other four subscales, the interpretations were as follows: a maximum of 24 points indicated extremely high stress, 18 indicated the preceptors were very stressed and 12 meant they were moderately stressed, while a value of six was linked to being slightly stressed.

The combined scores for the entire CSS were calculated by adding up all subscales and averaging them across the sample size. The maximum values of 144 for the combined scores indicated extremely high levels of stress, 108 indicated the preceptor was very stressed, 72 meant they were moderately stressed and 36 was linked to being slightly stressed, while zero meant no stress at all.

### Burnout Assessment Tool analysis

The subjective rating from each of the six categories of the BAT were summed by adding the respective scores for each of the items within the respective category. The averages of each of the categories were interpreted using the associated scale, which indicated the frequency that preceptors experience certain symptoms associated with burnout. One meant the preceptor never experienced burnout, two indicated burnouts to be a rare occurrence, three meant the preceptor sometimes experienced burnout, four indicated that burnout was experienced often and five showed the preceptor always experienced burnout.

For the qualitative data analysis, the recorded interviews were transcribed verbatim and analysed deductively through reading and coding (Saldaña 2021:138). The data were analysed on the ATLAS.ti (ATLAS.ti Scientific Software Development GmbH, Berlin, Germany) platform. The transcripts were coded using a combination of coding methods, namely initial, structural, in-vivo and open coding (Saldaña 2021:140). The authors discussed the coding outputs and agreed on the outcome of the initial analysis. Pattern coding was applied as a secondary coding approach influenced by the research question to support the mapping of the outcomes of the study with the preceptor support framework.

### Rigour and trustworthiness

The four measures of confirmability, transferability, dependability and credibility ensured trustworthiness (Creswell 2014:206; Shenton 2004:64). To ensure confirmability in the study, all researchers analysed the transcripts individually (Creswell 2014:203; Saldaña 2021:70). The researchers then met to compare and discuss the coding and reach consensus. The interviewer is an expert in qualitative research, which provided dependability and credibility to the data collection (Shenton 2004:67). Furthermore, credibility was ensured through the audit trail, which included audio-recording and verbatim transcription of the individual interviews with preceptors and a critical review by a research team member not involved in the interview process.

### Ethical considerations

Ethics approval was obtained from the Health Sciences Research Ethics Committee from the university (reference number UFS-HSD2021/0474/2707). Three ethical principles, namely beneficence and nonmaleficence, distributive justice and respect for persons were applied in this study (Creswell 2014:130). The authors minimised any potential risk of harm from this study and availed opportunities for psychological support after the interviews, and participants had an opportunity to express discomfort with a possibility of ending the interviews. All participants had an equal chance of being part of the study and any associated benefits such as psychological support post interviews. The authors respected the participants throughout the study, and the participants were provided with sufficient information to decide on being part of the study. Participants further had an opportunity to pull out of the study without consequence.

## Results

Eleven preceptors responded to the questionnaires, indicating a response rate of 45.83%. Oates (2012:99) indicates that a response rate of ≥ 30.00% per sample size is considered a good response rate for electronically administered questionnaires. Results of stress and burnout are presented before discussing the results integrated with the experiences of preceptor support outcomes mapped against the preceptor support framework.

### Levels of coronavirus disease 2019 stress

**FIGURE 2:**
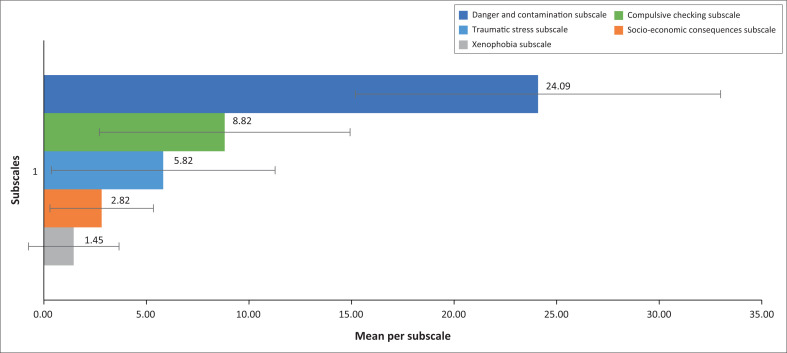
COVID-19 Stress Scale data with standard deviation.

The five subscales indicated the levels of stress experienced by preceptors during the COVID-19 pandemic. Figure 2 presents the outcome of the CSS from the respondents, with the respective standard deviation (s.d.) as error bars.

The overall data for each subscale indicated an average score varying from no stress to moderate stress, apart from some preceptors experiencing a very high level of stress, as can be seen by the standard deviations as high as 33 within the danger and contamination subscale.

### Levels of burnout

**FIGURE 3:**
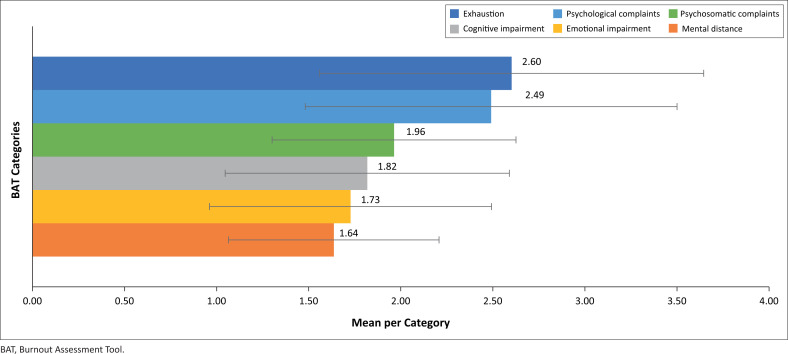
Burnout Assessment Tool data with standard deviation.

The BAT was used to determine the levels of burnout in six categories, based on respondents’ self-reported burnout. The averaged data, with their standard deviations as error bars per category as indicated in Figure 3, shows that the respondents rarely experienced burnout. However, some respondents experienced high levels of burnout, as indicated in the standard deviations as high as 3.6 within the exhaustion subscale.

### Preceptor support framework outcomes

The preceptor support framework is underpinned by three domains, namely system, emotional and cognitive support. The outcome of this study is reported by integrating excerpts from the semistructured interviews with the results from the CSS and BAT instruments mapped against the three domains of the preceptor support framework. Within each support domain, selected relevant items from the stress and burnout items are discussed.

#### System support

The system support domain is aligned with subscales of the CSS related to danger and contamination, socio-economic consequences, xenophobia, and compulsive checking. No BAT items were included.

Regarding the danger and contamination subscale, the preceptors scored a high score of 24.09 with a standard deviation of 8.9, indicating a moderately stressed score. This moderately high score related to stress is supported by the preceptors, who mentioned:

‘I will never go into the ward … Then they’ll [*the students*] come to me outside the wards. Then that’s where we will be holding … the discussions outside the wards.’ (P5, female, 08 April 2022)

‘My mother – I was, like, scared because she is also having some chronic illness, like diabetes. So I was scared that if […] she can get COVID-19 virus infection, it can be actually a very difficult situation for her.’ (P1, male, 09 April 2022)

In the socio-economic consequences, the respondents scored 2.82 with a standard deviation of 2.52, which indicates slightly stressed to no stress. The preceptors revealed that the socio-economic consequences had limited bearing on their system’s support function, as the CLE ensured that students are supplied with all necessary resources, albeit their shortage. In one of the interviews, a preceptor mentioned:

‘Also, the students were also supplied with some hand sanitisers and the PPE […] so that was also a lot, because […] during that time, remember, you also go to the shop, you go to the shop, you actually find that the shelves that are supposed to have gloves or sanitisers, it’s actually finish.’ (P1, male, 09 April 2022)

The xenophobia subscale score of 1.45, with a standard deviation of 2.21, indicates that the preceptors were not stressed in relation to foreign nationals transmitting the virus. This result is supported by the interview data, as none of the preceptors mentioned the influence of foreigners in their role as they supported students during the pandemic.

The final subscale of the CSS related to system support is the compulsive checking subscale, which was classified as slightly to moderately stressed, with a rating of 8.82 and a standard deviation of 6.11. The preceptors supported this outcome in the interviews regarding the amount of information in the media and website of the university directly influencing their supportive roles as they accompanied students. One preceptor mentioned:

‘There was too much in the media to read about COVID. Even if you know when you go to work, then they will be in service as trainees. Then you go to the website of the UFS [*University of the Free State*] then really that was also another information, so we were really….’ (P1, male, 09 April 2022)

#### Emotional support

The subscales of the CSS were mapped to the emotional support of the preceptor support framework, namely the danger and contamination, socio-economic consequences, compulsive checking and traumatic stress. In addition, three subscales of the BAT scale were mapped to emotional support, namely emotional impairment, psychosomatic complaints and exhaustion. Preceptors are expected to provide emotional support to students related to their clinical learning. One preceptor was of the opinion that:

‘I think more during COVID-19, it wasn’t really about the cognitive support or maybe more focusing on the academic aspect of preceptoring. It was more based on the emotional aspect, because the students are going through a lot.’ (P2, female, 07 April 2022)

As presented in the system support section, the danger and contamination subscale had resulted in moderately stressed scores. The semistructured interviews show that the preceptors also struggled to provide emotional support for students for fear of contracting the virus. The preceptors felt they had to strike a balance between protecting students and affording emotional support. One preceptor mentioned:

‘Especially wearing masks or so every time. So sometimes you’ll be talking to the students. They don’t hear you. You have. You don’t know now because there’s the mask. Here’s the masks. Now you have to extend our vocal cords for you as to hear each other now.’ (P5, female, 08 April 2022)

The preceptors scored between slightly and no stress in relation to socio-economic consequences. The preceptors appeared to be focused and worried about the socio-economic influence of the virus on the community and their support function with students. One of the preceptors mentioned:

‘But now the question also comes: how are you going to do this if the community that you are actually teaching with the students that you are teaching, they are also affected psychological cause nurses? At the end, we are there to comfort our patients. But then how do we comfort them if we are also dealing with our own problems on the other side, and the patients are also having problems on their side because we deal with the media existential state of vulnerability?’ (P1, male, 09 April 2022)

In the compulsive checking subscale, the outcome was slightly to moderately stressed. The influx of information related to the COVID-19 pandemic has a substantive influence on students and their emotional well-being, including their ability to engage with patient care and subsequent debriefing after caring for patients with COVID-19. The preceptors had to actively manage the situations by being role models for the students, thus creating an opportunity for psychological safety. One preceptor mentioned:

‘I must be a nurse though I accompany the students, but now I must also be a nurse because now we are all role models also to them. So if we fear, what about the students? That’s how I was coping. So I was trying to be strong for the students.’ (P5, female, 08 April 2022)

The outcome of the traumatic subscale is below the slightly stressed level, with a score of 5.82 and standard deviation of 5.46. The findings of the traumatic subscale chimed with those from the BAT score related to emotional impairment (1.73). The relatively high scores are supported by the preceptors’ experiences, as reported through the semistructured interviews. One preceptor mentioned:

‘It actually also affected our psychological functioning because we were living with anxiety and the fear of unknown of what’s going to happen.’ (P1, male, 09 April 2022)

‘Everybody was so stressed and mean, and the students were just angry with everybody all the time, and it was quite a challenge.’ (P4, female, 08 April 2022)

Psychosomatic complaints (1.96) were reported as between never to rarely experienced in burnout. Although preceptors did not disclose psychosomatic symptoms during the semistructured interview, they did notice symptoms among students they supported. One of the preceptors stated:

‘But you could sense the fear and the uncertainty was tangible in the class. And with that comes then severe attitude problem. The students became very – not aggressive but rude, and they wanted answers that we could not give. Because we didn’t have the answers ourselves. So this was very challenging for it – for everyone.’ (P4, female, 08 April 2022)

However, preceptors experienced a significant amount of the exhaustion evidence by a score of 2.60 and standard deviation of 1.40. The exhaustion could, to an extent, have influenced the amount of emotional support to students during the pandemic:

‘Actually, I’m getting tired. I’m exhausted. But now the duty calls. And you also want to be responsible for students. So you will for your own sanity. Again, you try to do those minor exercises indoor at home.’ (P1, male, 09 April 2022)

#### Cognitive support

Cognitive support focuses on the development of the thinking operations of the student towards attaining their learning outcomes (Hugo et al. 2018:87). Four subscales of the CSS instrument and three subscales in the BAT aligned with the cognitive support domain. These subscales are the danger and contamination, xenophobia, traumatic stress and compulsive checking subscales. Mental distance, cognitive impairment and psychological complaints from the BAT were mapped to the cognitive support domain.

As previously mentioned, the danger and contamination subscale reflected a moderately stressed score. From the preceptors’ comments, it was apparent that the fear of contracting COVID-19 and the danger it posed could have impacted the cognitive support to students:

‘And I felt that because of the social distancing and all, we didn’t have sufficient time to do our job, you know, you have to be very brief. You go in and demonstrate procedures, and you don’t have as much time as you used to with them before the pandemic. So for me, preceptoring during that time was very, very difficult.’ (P3, female, 08 April 2022)

‘It was the time factor … limited face-to-face interactions … you have to teach, but you have to be brave about it, you know … don’t want to be in clinical settings because … don’t want to contract COVID.’ (P3, female, 08 April 2022)

The xenophobia subscale outcomes were just above the ‘not at all stressed’ score and there were no indications of this subscale in any of the preceptors’ interviews. In the traumatic stress subscale, however, the outcomes were just below the ‘slightly stressed’ score; some preceptors indicated a higher level of traumatic stress, which could have influenced the support they offered to the students during accompaniment. One preceptor stated:

‘So that definitely did put extra stress on everyone, because your schedule was the whole time being shifted. And I know some of us are only part time, so our schedules clash with our other jobs and – or other responsibilities. So that definitely did make things a bit more difficult.’ (P4, female, 08 April 2022)

Regarding the compulsive checking subscale, the outcome was between the slightly and moderately stressed scores. The preceptors reportedly insisted that students implement measures and checks which are over and above that of the normal regulations. One of the preceptors said:

‘The sister cannot just call to check with the student only. So the medication was tricky and had to be checked by three people at the end.’ (P1, male, 09 April 2022)

The values for the BAT categories of mental distance (1.64), cognitive impairment (1.82) and psychological complaints (2.49) were between the ranges of rarely to sometimes experienced burnout. Mental distance was experienced by preceptors.

‘It was, but it was very, very difficult. We felt extremely isolated at home. And then when you did go to work, everybody was so stressed.’ (P4, female, 08 April 2022)

‘During that time of COVID, it was not easy. Because it was working in your mind, but now it’s like….’ (P5, female, 08 April 2022)

None of the participants reported cognitive impairment during their accompaniment of students in the CLE. However, a preceptor mentioned the psychological effect of having had to support students in the CLE during the pandemic:

‘It actually also affected our psychological functioning, because we were living with anxiety and the fear of unknown of what’s going to happen. So that was also a difficult situation for us as facilitators.’ (P1, male, 09 April 2022)

## Discussion

The COVID-19 pandemic had a direct and indirect influence on the mental health of preceptors, affecting their role in supporting students in the CLE. Professional development programmes for preceptors often focus on the competence development of individuals as preceptors with limited focus on strategies towards enhancing resilience among preceptors during adversities such as the COVID-19 pandemic. In this study, a mixed methods approach underpinned by the preceptor support framework was used to explore the influence of the COVID-19 pandemic on the role of preceptors in supporting students at an NEI. The overall aim was to measure stress and burnout, which gives insights into what should be included in the professional development programmes for preceptors to enhance their resilience and supportive role during times of adversity.

System support requires preceptors to communicate pertinent information to healthcare professionals in the CLE, such as students’ workload, learning outcomes and a space to conduct facilitation while establishing an active role within the team for students in patient care activities (Hugo et al. 2018:87). This study showed that the system support role of preceptors was affected by the COVID-19 pandemic. The preceptors preferred not to enter the CLE for fear of contracting and transmitting the COVID-19 virus. In this setting, the limited telecommunication infrastructure and minimal integration of telecommunication in healthcare imply that preceptors had to execute their system support role by physically communicating information related to students in the CLE. Lee (2020:393) stated that mass tragedies, particularly ones that involve infectious disease, often trigger waves of heightened fear and anxiety, causing massive disruptions to the behaviour and psychological well-being of many in the population. Healthcare professionals, in general, have a higher propensity to contract the COVID-19 due to their occupational risk, thus increasing their emotional reaction to the virus. Troisi et al. (2021:1) explain that the fear of contracting the virus among health professionals contributes to occupational inefficiency with ripple effects. In this study, the suboptimal system support offered to students in the CLE by preceptors may have contributed to students’ distress and lack of clarity in their role and function in the CLE, as observed by the preceptors in some students.

Research has revealed that a lack of PPE contributed to moral distress among healthcare professionals, heightening their fear of contracting the virus (O’Neal et al. 2021:2). However, contrary to reports from South Africa and other regions, the students and preceptors in this setting received sufficient PPE. The availability of PPE for students and preceptors diluted the fears of the preceptors in rendering system support. Preceptor professional development programme planners must identify fears among preceptors related to adversity and integrate strategies that dilute the fears of preceptors as part of the professional development programme. Suppressed fears may enhance the efficiency of preceptors in rendering support to students (Wiemer et al. 2021:221).

Emotional support entails an interpersonal relational building approach where preceptors are interested in students, knowing and encouraging them while building their confidence in patient care activities (Hugo et al. 2018:87). Preceptors regarded emotional support as an essential type of support for nursing students during the COVID-19 pandemic. In this study, the preceptors exhibited empathy and compassion towards the students in the CLE. Empathy aligns with vicariously identifying with the feelings of other people, while compassion is rooted in the desire to help (Stevens & Taber 2021:2). Preceptors in this study continuously engaged students emotionally and used themselves as role models for students. Role modelling of empathy and compassion by nurse educators has been shown to have a positive impact on students’ professional development as nurses. Empathy and compassion demonstrated by the preceptors in this study are essential attributes that support the emotional growth and development of nursing students (Straughair 2012:239).

Preceptors reported feeling exhausted during the COVID-19 pandemic as they executed their supportive role. The integration of multiple factors, including role modelling empathy and compassion, may justify the experiences related to exhaustion. Exhaustion negatively affects work performance (Burki 2020:1402). Giauque et al. (2022:21) added that in times of exhaustion, individuals’ level of collaboration and engagement declines. Exhaustion and burnout have been reported among preceptors in Australian paediatric settings (Frankenberger et al. 2021:1), which were linked to cognitive strain and the lack of preparation for the role. Preceptors in this study may not have been adequately prepared to provide emotional support for students during the COVID-19 pandemic. Emergency measures to engage and salvage the academic programme instituted by NEIs ignored the preparation of preceptors for emotional support for students during the pandemic. Furthermore, the lack of debriefing strategies for preceptors by professionals such as clinical psychologists may have contributed to exhaustion. Preceptor professional development programmes must integrate strategies that support preceptors in role modelling empathy and compassion but at the same time create avenues that allow for their preparation for their roles in adversity and opportunities for debriefing.

Cognitive support is an important support domain where preceptors use various facilitation techniques to support students’ learning to enhance competence. Students are guided to notice and interpret patient information, respond appropriately to patients’ healthcare needs and reflect on care given (Hugo et al. 2018:87; Tanner 2006:208). The complexity of nursing management activities increased during COVID-19 as treatment and guidelines for care were constantly being revised. The preceptors described heightened compulsive checking of information related to the pandemic from various sources. The advent of such a novel virus and the expansion of electronic sources of information created opportunities for the circulation of false information, which, if accessed by preceptors, would have directly influenced their abilities to offer cognitive support. Chong et al. (2020:1) cautioned against healthcare workers being at the forefront of rapidly disseminating premature information, which had a catastrophic effect during the COVID-19 pandemic. Inasmuch as the heightened compulsive checking of information by preceptors in this study is a positive outcome, preceptors must be aware of credible sources of information and should have the ability to critically appraise such information to enhance their cognitive support for nursing students.

Structural issues such as scheduling, social distancing and PPE had a direct influence in the process of cognitive support. The CLE was unstable, influenced by the peaks in COVID-19 transmission rates within the society, admissions into the hospitals and the suitability of physical infrastructure (O’Keefe & Auffermann 2022:S61). Schedules for students and preceptors were constantly changing, resulting in inadequate approaches for cognitive support. In addition, physical distancing requirements meant that preceptors would not provide the essential contact that contributes to learning. The literature reports various strategies that had been integrated to augment the limited physical contact issues associated with COVID-19. Clinical education via telemedicine, online clinical education including livestreaming of care, integrating standardised patients, clinical case-based scenarios and modified onsite clinical education are examples of approaches to augment the limitations of clinical education during the COVID-19 pandemic (Park, Shimm & Lee 2021:964). Preceptor development programmes must integrate innovative approaches that engage technology in clinical education to enhance their cognitive supportive role. Consideration of available resources, feasibility and the tech-savviness of the preceptors would be essential in tailor-making approaches for integrating innovation in their professional development programme.

This single-site mixed methods study is limited by the low number of preceptors who participated in both arms of the study. However, the study outcomes may resonate with other NEIs in South Africa, inasmuch as the descriptive aims of the study were focused on presenting contextual outcomes.

## Conclusion

The COVID-19 pandemic influenced the role of preceptors in supporting students in the CLE, as they had to amend their functioning role significantly while providing system, emotional and cognitive support. In this study, the preceptors experienced fear and anxiety in their role when they accompanied students but exhibited signs of resilience as they continued their supportive role amid a state of emergency. In times of adversity, emotional support is perceived as more important than cognitive support, as seen from participants’ responses. Nursing education institutions should provide professional development programmes that integrate concepts related to resilience to equip preceptors to function in adverse circumstances.

Existing preceptor professional development programmes should be reviewed to ensure that the necessary concepts that foster resilience are integrated to enhance the functional role of preceptors in adversity. The following concepts must be considered:

- The identification of fears among preceptors related to adversity and the integration of practical strategies that dilute the fears.
- The integration of strategies that support preceptors in role modelling empathy and compassion, at the same time creating avenues for preparation of their roles in adversity and opportunities for debriefing.
- The process of identifying credible sources of information and approaches towards critical appraising of health information.

Preceptors play a critical role in supporting nursing students in the CLE. Education programmes for developing preceptors must be responsive and prepare the preceptors to be mentally resilient during adversity. Not every clinician is a good preceptor, but the best preceptors are often trained and supported in executing their role.
